# Insertion of an immunodominant T helper cell epitope within the Group A *Streptococcus* M protein promotes an IFN-γ-dependent shift from a non-protective to a protective immune response

**DOI:** 10.3389/fimmu.2023.1241485

**Published:** 2023-08-15

**Authors:** Shiva Emami, Thiago Rojas Converso, Jenny J. Persson, Bengt Johansson-Lindbom

**Affiliations:** Immunology Section, Department of Experimental Medical Science, Biomedical Center, Lund University, Lund, Sweden

**Keywords:** group A *Streptococcus*, T cells, B cells, IFN-γ, Antibodies, IgG2c, M protein, protection

## Abstract

The common pathogen Group A *Streptococcus* (GAS, *Streptococcus pyogenes*) is an extracellular bacterium that is associated with a multitude of infectious syndromes spanning a wide range of severity. The surface-exposed M protein is a major GAS virulence factor that is also target for protective antibody responses. In this study, we use a murine immunization model to investigate aspects of the cellular and molecular foundation for protective adaptive immune responses generated against GAS. We show that a wild type M1 GAS strain induces a non-protective antibody response, while an isogenic strain carrying the immunodominant 2W T helper cell epitope within the M protein elicits an immune response that is protective against the parental non-recombinant M1 GAS strain. Although the two strains induce total anti-GAS IgG levels of similar magnitude, only the 2W-carrying strain promotes elevated titers of the complement-fixing IgG2c subclass. Protection is dependent on IFN-γ, and IFN-γ-deficient mice show a specific reduction in IgG2c levels. Our findings suggest that inclusion of the 2W T cell epitope in the M protein confers essential qualitative alterations in the adaptive immune response against GAS, and that sparsity in IFN-γ-promoting Th cell epitopes in the M protein may constitute an immune evasion mechanism, evolved to allow the pathogen to avoid attack by complement-fixing antibodies.

## 1 Introduction

Group A *Streptococcus* (GAS) is a prevalent human pathogen causing more than 700 million cases of mild infections and around 500.000 deaths annually worldwide (1, 2). It is responsible for a wide spectrum of diseases ranging from mild infections, such as pharyngitis and impetigo, to severe life-threatening conditions like necrotizing fasciitis and streptococcal toxic shock syndrome (STSS). In addition, in the aftermath of recurring mild GAS infection, the severe auto-immune complication acute rheumatic fever (ARF) may develop (3). Of note, the serious and historically significant GAS-associated disease scarlet fever, which over the past century has declined remarkably in prevalence, has for unknown reasons re-emerged as a relatively common syndrome in some locations during the last decade (4–7).

GAS express an impressive array of virulence factors, likely contributing to the ability of this pathogen to establish infection in virtually any tissue (8). Among its most significant virulence factors is the surface-exposed M protein, a fibrillar, multi-functional protein that is expressed at high density on the bacterial surface (9) and has been ascribed major importance in GAS pathogenesis (10). The most well-studied and best understood function of the M protein is its ability to confer phagocytosis resistance, although several additional roles have been stipulated (10–12). The N-terminal of M proteins contains a hypervariable region (HVR) that exhibits significant variability between strains, and allows for the identification of >220 different *emm* (or M) types (13). Importantly, the HVR is also target for type-specific protective antibodies while exhibiting immuno-subdominance, thus allowing GAS strains to escape antibody attack trough both antigenic variation and weak immunogenicity (14, 15). Despite decades of research, there is no licensed vaccine against GAS. The challenges facing GAS vaccine development are many, including the above-mentioned extensive strain variability and immune-subdominant characteristics of known protective epitopes. In addition, antibodies generated against GAS M proteins may in rare cases cause autoimmune responses towards highly similar host proteins, *e.g.* cardiac myosin, causing the post-streptococcal syndrome ARF which may progress to the severe condition rheumatic heart disease (16). Indeed, vaccine research on GAS was banned by the FDA for 30 years after a catastrophic American trial in the 1960’s where a number of participating children developed ARF after vaccination with an M protein-based vaccine (17, 18). Currently however, several M protein-based candidates are again assessed in early human trials (19), and an increased understanding of how M protein-targeted immune responses mediate protection should facilitate the further development of vaccines that are fully or partially based on this GAS protein family.

Although it remains unclear how IFN-γ regulates selection of isotypes and IgG subclasses in the human system, it has been suggested that high IFN-γ levels correlate with anti-GAS antibodies of the opsonizing and complement-fixing IgG3 and IgG1 subclasses, and contribute to enhanced immunity against GAS in adults as compared to children (20). Similarly, in patients with the tick-borne infection Lyme borreliosis, high levels of IFN-γ correlate with a switch to IgG3 and protection from chronic disease development (21). In several murine GAS infection models, IFN-γ has been implicated in protective immunity (22, 23). Murine antibodies of the IgG2a/c subclass (IgG2a in BALB/c, IgG2c in C57Bl/6 (B6) mice) functionally correspond to the human complement-fixing subclasses IgG3 and IgG1 and are mainly driven by IFN-γ (24, 25). In contrast, murine IgG3 and IgG1 antibodies show no or limited capacity to fix complement (26).

In addition to humoral immunity, GAS induces CD4 T cell responses (27–29) likely to contribute to protection both through provision of B cell help and through effects on neutrophils and macrophages. During primary immunization, naïve CD4 T cells recognize foreign peptides in complex with MHC class II molecules (p:MHCII) presented on the surface of antigen-presenting cells. This interaction leads to clonal T cell expansion and, depending on local cues, differentiation into polarized cytokine-producing T helper cell (Th) cell subsets, including IFN-γ producing Th1 cells, IL-17-producing Th17 cells and T follicular helper (Tfh) cells; the latter specialized in assisting clonal B cell expansion and affinity maturation within germinal centers (GCs) (30, 31). Studies of endogenous *in vivo* polyclonal antigen-specific Th cell responses have been facilitated by the p:MHCII tetramer technology, where soluble and biotinylated p:MHCII complexes are multimerized by fluorescently labeled streptavidin, allowing detection of rare endogenous p:MHCII specific CD4 T cells by flow cytometry (32). The technology relies on the identification of pathogen-derived peptides that form immunodominant p:MHCII epitopes together with the MHCII alleles expressed by the host. GAS-derived peptides forming such immunodominant epitopes with the I-A^b^ MHCII molecules of B6 mice have however not yet been identified. To study endogenous polyclonal Th cell responses against GAS, a recombinant M1 strain has instead been generated, containing the 2W variant of peptide 52-68 from the MHCII I-E α-chain inserted in the N-terminal of the M1 protein (GAS-2W.M1) (29, 33). In B6 mice, lacking I-E molecules, the precursor frequency of naïve CD4 T cells recognizing 2W:I-A^b^ is very high and this is linked to one of the highest p:MHCII-specific response magnitudes so far detected in this mouse strain (34–36). Consistent with this, intranasal inoculation with GAS-2W.M1 leads to a robust expansion of 2W:I-A^b^-specific CD4 T cells, which develop into IL-17-producing cells that clear bacterial carriage in the nasal mucosa (29). Following subcutaneous (sc) or intravenous immunization, the expanded 2W:IA^b^ specific T cells however fail to produce IL-17 and instead develop into IFN-γ producing Th1 cells (29).

In the current study, we demonstrate that sc administration of heat-killed (HK) GAS of the M1 serotype (GAS-M1) elicits significant IgG responses but fails to induce protective immunity in B6 mice. Protection against GAS-M1 is however achieved following immunization with the mutant GAS-2W.M1 strain. Using mice lacking Tfh cells and IFN-γ deficient mice, we further demonstrate that protection relies on a T cell-dependent antibody response and IFN-γ. Interestingly, mice immunized with GAS-2W.M1 show similar levels of total IgG against GAS-M1 as GAS-M1-immunized mice but display a selective increase in antibodies of the IgG2c subclass. Collectively, this indicates that following sc GAS immunization the, 2W:I-A^b^ Th cell epitope induces an IFN-γ response that confers protection by altering the isotype composition of the antibody response against the bacteria.

## 2 Materials and methods

### 2.1 Bacterial strains and culture conditions

The GAS strain 90-226 is an M1 serotype strain originally isolated from a sepsis patient (37), and is herein referred to as GAS-M1. GAS-2W.M1 is a genetically engineered strain in which the 14 amino acid peptide 2W (EAWGALANWAVDSA) has been inserted in frame after the first five amino acids of the mature M1 protein of strain 90-226 (29). All strains were grown without shaking in Todd-Hewitt broth supplemented with 0.2% yeast extract (THY) in 5% CO_2_ at 37^°^C.

### 2.2 Mice

Female C57Bl/6 (wildtype, [B6]) mice were purchased from Taconic and IFN-γ-KO (B6.129S7-Ifng^tm1Ts^/J) mice (generated on 129S7/SvEvBrd-*Hprt1^+^*, backcrossed 8 generations on C57BL/6) were originally from The Jackson Laboratory. CD4-Cre (B6.CgTg(Cd4-cre)1Cwi/BfluJ) and Bcl6^fl/fl^ (B6.129S(FVB)Bcl6 ^tml.1Dent^/J) mice, both on B6 background, were crossed to generate CD4-Cre x Bcl6^fl/fl^ animals lacking the T follicular helper (Tfh) cell master transcription factor Bcl6 specifically in CD4^+^ T-cells. For experiments using genetically modified mice, both females and males were used. Experiments were initiated at animal ages of 8 – 12 weeks. All animals were bred (except B6) and maintained at the animal facility at the Lund University Biomedical Center, and experiments were performed in accordance with protocols approved by the Lund/Malmö Animal Ethics Committee.

### 2.3 Immunizations and infections

Mice were immunized using 10^8^ heat-killed (HK) GAS-M1 or GAS-2W.M1. Bacterial over-night cultures were re-inoculated and grown to OD= 0.8 - 1, after which cultures were washed in PBS and incubated for 2 hours at 60˚C. HK cultures were diluted to appropriate concentration, aliquoted and stored in -20°C. Complete bacterial killing was confirmed by plating an aliquot on blood agar for counting. Mice were injected subcutaneously (sc) three times with three-week intervals and bled 19 days after each injection. For protection experiments, a lethal dose of 10^8^ CFU GAS-M1 was distributed intraperitoneally (ip) three weeks after the last immunization injection. Infected mice were monitored every four hours for the first day and then twice daily for one week. After infection, cages were blinded to avoid bias in determining moribundity.

### 2.4 Serum antibody titer measurements

To determine antibody titers in murine serum, HiBond ELISA plates were coated with whole GAS-M1 bacteria, or recombinant M1 protein or isolated M1 HVR [generated as described in (38)] and incubated with serially diluted serum. Captured antibodies were detected using biotinylated goat anti-mouse IgG (Southern Biotech, CAT 1036-08), IgG1 (BioLegend, RMG1-1), IgG2b (BioLegend, RMG2b-1), and IgG2c (Southern Biotech, CAT 1079-08) and streptavidin-HPR (BioLegend, CAT 405210). BSA-coated wells served as negative controls and presented values represent the average of duplicate experimental values with deducted negative control values. All samples were run in duplicate.

### 2.5 Antibodies and reagents

Anti-CD16/CD32 (BD, 2.4G2 [anti-FcγR]) was used as a blocking reagent and PI-PECF594 (Invitrogen, CAT P3566) was used to identify dead cells. The following antibodies and reagents were used for germinal center B-cell staining: anti-IgD-BV-605 (BioLegend,11-26c.2a), anti-B220-APCeFlour780 (eBioscience, RA3-6B2), anti-CD38-Alexa 700 (eBioscience, 90), and anti-CD95-PE-CY7 (BD, Jo2). For T follicular helper cell (Tfh) staining, anti-CXCR5 (BD, 2G8), anti-rat IgG (fab’) Alexa-647 (Jackson Immuno Research, Code:712-606-153). 2W-specific T-cells were identified using a 2W:I-Ab tetramer labeled with PE (50959 I-A(b)) or BV421(50960 I-A(b) (NIH Tetramer Core Facility), and the following antibodies: anti-CD3-Alexa flour 700 (BioLegend, 17A2), anti-CD4-FITC (BioLegend, RM4-5), anti-B220-APCeFlour780 (BioLegend, RA3-6B2), and anti-PD1-PE-CY7 (eBioscience, J43). The Click-iT™ Plus EdU Alexa fluor 647 Flow cytometry kit was used for EdU staining of newly synthesized DNA (Invitrogen, CAT C10634). Porcine cardiac myosin (Sigma-Aldrich, CAT: M0531) and a cardiac myosin monoclonal antibody (ThermoFisher, CAT: MA1-26180) was used for investigation of myosin-reactive antibody responses.

### 2.6 Cell preparation and flow cytometry

Single cell suspensions were prepared by mechanical disruption of inguinal lymph nodes, and filtering of cells through a 70 μM strainer. The total number of white blood cells per lymph node was determined in a Sysmex hematology analyzer (Sysmex) and divided into two parts for detection of T cells and germinal center B cells. Cell suspensions were incubated with anti-FcγR in 10% rat serum to minimize Fc receptor-dependent background staining, after which the appropriate antibody mix was added. Dead cells were identified using PI-PECF594. For tetramer staining of 2W-specific T cells, cells positive for both tetramers were considered 2W-specific. Intracellular IFN-γ was detected in T cells stimulated with PMA, ionomycin and brefeldin A.

### 2.7 Statistical analysis

Data were analyzed with Prism version 8.0 (GraphPad Software). Analysis of statistical significance was performed using one-way ANOVA with Tukey’s multiple comparisons test for three or more groups, or Mann-Whitney U test for two unpaired groups. Comparison of Survival Curves was done by Kaplan-Meier survival test with Log-rank (Mantel-Cox) test to evaluate p value. Differences were considered significant when p ≤ 0.05.

### 2.8 Study approval

All experiments conducted were approved by the Ethical Committee for Laboratory Animals in Lund/Malmö, permit numbers: 7342/2017 and 07178/2020.

## 3 Results

### 3.1 Lack of protective immunity after GAS-M1 immunization is surmounted by an N-terminal insertion of the immunodominant CD4 Th cell epitope 2W into the M1 protein

**Figure 1:**
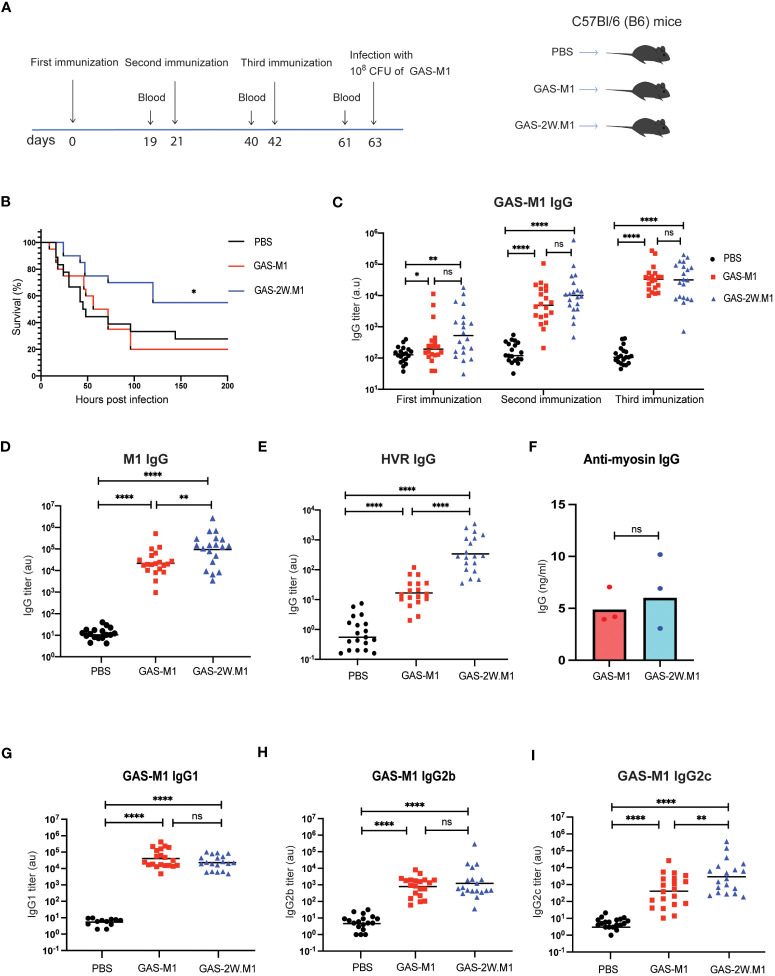
Immunization with GAS-2W.M1 induces protective immunity against the parental GAS-M1 strain. B6 female mice received three sc doses of HK GAS-M1, GAS-2W.M1or PBS with three weeks intervals (n=20 per group). Blood was collected for serum antibody analysis 19 days after each immunization (2 days before subsequent dose). Three weeks after third dose, mice were challenged ip with 10^8^ CFU of live GAS-M1 and then monitored for 7 days. **(A)** Experimental study design. **(B)** Survival after the lethal challenge, analyzed by Kaplan-Meier and Log-Rank test between control and each immunized group. **(C)** Serum IgG titers against intact GAS-M1 bacteria after the first, second and third dose. **(D, E)** Serum anti-M1 **(D)** and anti-HVR **(E)** IgG titers after the third immunization. **(F)** Anti-cardiac myosin IgG concentrations after the third immunization. **(G–I)** IgG subclass analyses, showing titers of IgG1 **(G)**, IgG2b **(H)** and IgG2c **(I)** against intact GAS-M1 bacteria after the third dose. Pooled results from three independent experiments with individual mice and geometric mean values are shown **(C–E, G–I)**. Anti-cardiac myosin IgG ELISA was done on pooled sera of all mice from indicated experimental group and results show three individual experiments and geometric mean concentrations **(F)**. Analysis of statistical significance was done using one-way ANOVA with Tukey’s multiple comparisons test. *p<0.05, **p<0.01 and ****p<0.0001. au, arbitrary units; ns, not significant.

B6 female mice were immunized subcutaneously (sc) with 10^8^ heat-killed (HK) bacteria (**Figure S1**), using either the strain 90-226 (serotype M1; GAS-M1) or the genetically engineered 90-226 strain variant, expressing a chimeric M1 protein with the immunodominant CD4 Th cell 2W epitope inserted in-frame after the first five amino acids of the mature M1 surface protein (GAS-2W.M1) (29). Control mice received PBS only. All mice were immunized three times with three weeks intervals and then injected intra-peritoneally (ip) with a lethal dose of wt GAS-M1 (10^8^ CFU) three weeks after the last immunization (**Figure 1A**). Thereafter the animals were monitored for seven days. Consistent with the results of Lannergård et al. (15), repeated immunization with wt GAS-M1 did not result in protective immunity, and mice subjected to this treatment exhibited equally poor survival to the lethal challenge as control mice receiving PBS only (**Figure 1B**). In contrast, immunization with GAS-2W.M1 resulted in a significantly enhanced survival following subsequent challenge with GAS-M1 – *i.e.* the strain not containing the 2W epitope (**Figure 1B**).

To assess if the enhanced survival of mice immunized with the recombinant GAS-2W.M1 strain correlated with enhanced antibody response against the parental GAS-M1 strain, blood was collected 19 days after each immunization, and sera were assayed for reactivity against intact GAS-M1 bacteria, the M1 protein, and the M1 HVR domain. While levels of IgG against intact GAS-M1 did not differ between mice immunized with GAS-M1 or GAS-2W.M1 (**Figure 1C**), levels of anti-M1 and anti-HVR IgG were higher in the GAS-2W.M1 immunized animals (**Figures 1D, E**). The post-GAS-infection autoimmune complication acute rheumatic fever (ARF) is believed to involve cross-reactive immune reactions between the M protein and cardiac myosin (39, 40). The ability of the GAS-2W.M1 strain to enhance IgG responses against the M protein and its HVR was however not associated with increased levels of IgG against cardiac myosin (**Figure 1F**), indicating that incorporation of the 2W Th cell epitope into the M protein does not unleash such autoimmune responses. Finally, we assessed the levels of distinct IgG subclasses against intact GAS-M1. We found no difference in IgG1 or IgG2b between the two immunized groups (**Figures 1G, H**), however the geometric mean IgG2c titer was significantly higher in GAS-2W.M1- than in GAS-M1-immunized animals (**Figure 1I**).

Altogether, these results show that insertion of the CD4 Th cell epitope 2W into the N-terminal part of the M1 protein gives rise to a recombinant strain with an enhanced ability to induce protective immunity against the parental wild type (wt) GAS-M1 strain, and that this protection is associated with increased IgG responses against the M1 protein as well as a more IgG2c-biased response against the bacteria.

### 3.2 GAS-2W.M1 immunization does not protect against GAS-M1 infection in the absence of T cell-dependent B cell responses

**Figure 2:**
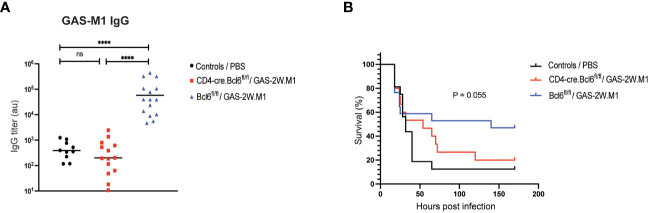
GAS-2W.M1 immunization fails to elicit protective IgG responses in Tfh cell deficient CD4-Cre.Bcl6^fl/fl^ mice. CD4-Cre.Bcl6^fl/fl^ (n=17) or Bcl6^fl-fl^ (n=15) mice received three sc doses of HK GAS-2W.M1 as described in **Figure 1**. A third group of mice (mix of both genotypes; n=15) received 3 x PBS only and served as unimmunized controls. Three weeks after the last immunization, mice were challenged ip with a lethal dose of live GAS-M1 (10^8^ CFU). Blood was 2 days before the lethal dose for serum antibody analysis and animals were monitored for 7 days post infection. **(A)** Serum IgG titers against intact GAS-M1 bacteria. Results show individual mice and geometric mean titers. Analysis of statistical significance was done using one-way ANOVA with Tukey’s multiple comparisons test. **(B)** Survival after the lethal challenge, analyzed by Kaplan-Meier and Log-Rank test between control and each immunized group. All results are pooled from three individual experiments. ****p<0.0001. au, arbitrary units; ns, not significant.

To determine if the ability of GAS-2W.M1 to confer protection against the parental GAS-M1 strain involves T cell-dependent antibody responses, we used CD4-Cre.Bcl6^fl/fl^ mice with a specific deletion of the transcriptional repressor Bcl6 in T cells. In these mice, germinal centers (GC) and associated high-affinity antibody responses fail to develop due to impaired T follicular helper (Tfh) cell development (41, 42). While Bcl6^fl/fl^ littermate controls (Cre^-^) developed robust anti-GAS-M1 IgG responses after GAS-2W.M1 immunization, CD4-Cre.Bcl6^fl/fl^ mice completely failed to do so (**Figure 2A**), demonstrating that the IgG response elicited by GAS-2W.M1 immunization is T cell-dependent. In subsequent survival experiments, immunized CD4-Cre.Bcl6^fl/fl^ mice were equally sensitive to the lethal dose of GAS-M1 as unimmunized littermates (**Figure 2B**). Compared to both of these groups, immunized Cre^-^ control mice displayed increased survival (**Figure 2B**). Although this difference did not quite reach significance (p=0.055), collectively the results indicate that T cell-dependent antibodies contribute to the overall protection induced against GAS-M1 following immunization with GAS-2W.M1.

### 3.3 The 2W Th cell epitope promotes an enhanced CD4 T cell IFN-γ response in GAS-2W.M1-immunized mice

**Figure 3:**
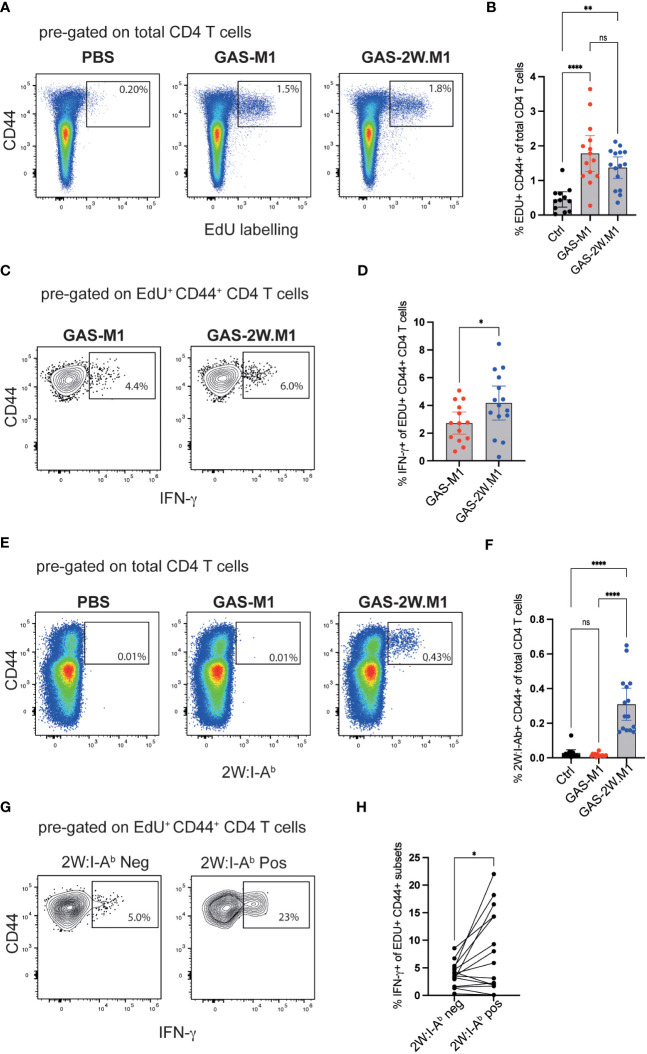
GAS-2W.M1 induces an enhanced CD4 T cell IFN-γ response relative to the parental GAS-M1 strain. B6 mice were immunized three times with HK GAS-M1 (n=15) or HK GAS-2W.M1 (n=14) as described in **Figure 1**. Control B6 mice received 3 x PBS only (n=12). Two days after the last immunization, EdU was injected ip and CD4 T cell responses in the draining inguinal lymph nodes were analyzed 2 days after the EdU pulse, following a brief *ex vivo* restimulation with PMA and ionomycin. Representative analyses and pooled results from three individual experiments are shown. **(A, B)** Percentage of CD44^+^ EdU^+^ cells after gating on live CD4^+^ B220^-^ T cells. **(C, D)** Percentage of IFN-γ^+^ cells of live EdU^+^ CD44^+^ CD4^+^ B220^-^ T cells. **(E, F)** Percentage CD44 + 2W:I-A^b+^ cells after gating on live CD4^+^ B220^-^ T cells. **(G, H)** Percentage IFN-γ^+^ cells among the 2W:I-Ab tetramer positive and negative subsets in GAS-2W.M1-immunized mice after gating on EdU^+^ CD44^+^ CD4^+^B220^-^ T cells. Individual mice and geometric mean values with one-way ANOVA Tukey’s multiple comparisons tests **(B, F)**, unpaired t-test **(D)** or paired t-test analyses **(H)** are shown. *p<0.05, **p<0.01, ****p<0.0001. ns, not significant.

Children are more susceptible to superficial GAS infections than adults and this correlates with reduced IgG3 and IFN-γ production (20). In mice, IFN-γ is promoting IgG2c responses, and given the increased IgG2c response observed after GAS-2W.M1 immunization (see **Figure 1I**), we next set out to compare IFN-γ production by GAS-specific CD4 T cells in mice immunized with GAS-M1 or GAS-2W.M1. B6 mice were again injected three times with PBS or HK bacteria (GAS-M1 or GAS-2W.M1). To track the CD4 T cells specifically responding to the immunizations, all mice received the thymidine analogue ethynyl deoxyuridine (EdU) via ip injection two days after the third immunization, which specifically labels all cells undergoing cell division during the *in vivo* pulse period (43). Two days after the EdU injection, mice were sacrificed and cells from draining inguinal lymph nodes were analyzed by flow cytometry following a brief *ex vivo* stimulation with PMA and ionomycin. The percentage of CD4 T cells that had incorporated EdU during the two-days pulse period was ~3-4 fold higher in mice immunized with HK bacteria relative to mice receiving PBS only, demonstrating that labelling with EdU to a high extent reports CD4 T cells proliferating in response to the GAS immunizations (**Figures 3A, B**). There was however no significant difference in EdU labelling between the GAS-M1 and the GAS-2W.M1 groups, indicating that insertion of 2W into the M1 protein does not lead to an enhanced overall expansion of GAS-specific CD4 T cells. Next, we focused on IFN-γ production by the CD4 T cells responding to the bacterial immunizations by gating on CD4^+^CD44^+^ (*i.e.* activated effector and memory) T cells with detectable EdU labelling. Among the EdU-labelled CD4^+^CD44^+^ T cells, a significantly higher percentage of cells produced IFN-γ in mice immunized with the GAS-2W.M1 strain as compared to mice immunized with GAS-M1, lacking the 2W epitope (**Figure 3C, D**). To determine if the 2W epitope itself contributes to this enhanced IFN-γ response, we used a 2W peptide-MHCII (2W:I-A^b^) tetramer to identify 2W-specific CD4 T cells (29). As expected, CD4 T cells binding to the 2W:I-A^b^ tetramer were present in mice immunized with GAS-2W.M1 but not detected in mice immunized with GAS-M1 or mice receiving PBS only (**Figures 3E, F**). Focusing on the GAS-2W.M1 immunized animals only, we next compared IFN-γ production by 2W:I-A^b^ positive and negative CD4^+^CD44^+^ T cells, respectively, also labelled by EdU. This approach allowed us to directly compare the 2W:I-A^b^-specific Th cells with activated effector and memory Th cells responding to peptide:MHCII complexes containing GAS-derived peptides distinct from the 2W epitope (derived from M1 or other GAS proteins). As shown in **Figures 3G** and **H**, the percentage of IFN-γ producing cells was significantly higher among the 2W:I-A^b^-positive than the 2W:I-A^b^-negative subset. Taken together, these results suggest that incorporation of the 2W Th cell epitope into the M1 protein generates a GAS-M1 strain that drives enhanced IFN-γ responses, as compared to its isogenic parental strain.

### 3.4 Failure of GAS-2W.M1 to induce protective immunity in IFN-γ deficient mice is associated with a specific loss in IgG2c responses

**Figure 4:**
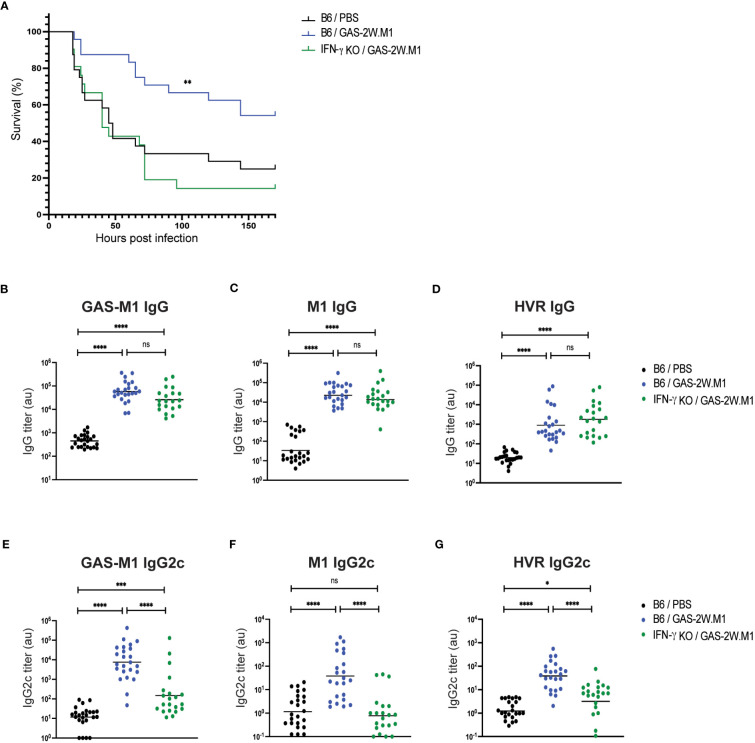
Lack of protective immunity and IgG2c responses in IFN-γ deficient mice following GAS-2W.M1 immunization. IFN-γ deficient (n=21) or sufficient (n=24) B6 mice were immunized sc three times with HK GAS-2W.M1 as described in **Figure 1**. Control B6 mice received 3 x PBS only. Blood was collected for serum antibody analysis 2 days before ip injection of a lethal dose GAS-M1 (10^8^ CFU), after which animals were monitored for 7 days. Results are pooled from three separate experiments. **(A)** Survival after the lethal challenge, analyzed by Kaplan-Meier and Log-Rank test between control and each immunized group. **(B–D)** Serum IgG titers against intact GAS-M1 bacteria **(B)**, M1 **(C)** and the M1 HVR **(D)**. **(E–G)** Serum IgG2c titers against intact GAS-M1 bacteria **(E)**, M1 **(F)** and the M1 HVR **(G)**. For measurement of antibodies against whole bacteria, GAS-M1 killed through treatment with 0.05% sodium azide was used for coating. Results show individual mice and geometric mean titers. Analysis of statistical significance was done using one-way ANOVA with Tukey’s multiple comparisons test. *p<0.05, **p<0.01, ***p<0.001 and ****p<0.0001. au, arbitrary units. ns, not significant.

Next, to assess if IFN-γ is important for the protection conferred by GAS-2W.M1 immunization, we used IFN-γ deficient mice. Mice were again immunized sc three times with HK GAS-2W.M1. Following lethal challenge with GAS-M1, IFN-γ deficient mice displayed a significantly reduced survival as compared to immunized IFN-γ sufficient mice (**Figure 4A**). Despite reduced survival in the absence of IFN-γ, GAS-2W.M1 immunization resulted in equally robust total IgG responses in the IFN-γ deficient and sufficient animals; evident when measuring IgG binding to whole bacteria, the M1 protein, or to the M1 HVR domain (**Figures 4B–D**). In marked contrast, IgG2c responses against GAS-M1, including those targeting the M1 protein and its HVR domain, were strongly reduced in the IFN-γ deficient mice (**Figures 4E–G**). Altogether, these results show that GAS-2W.M1 immunization confers protection against the parental GAS-M1 strain through an IFN-γ-dependent mechanism, and that reduced survival in the absence of IFN-γ production is associated with a specific loss in anti-bacterial IgG2c responses.

### 3.5 IFN-γ deficiency does not restrain germinal center B cell or Tfh cell responses following GAS-2W.M1 immunization

**Figure 5:**
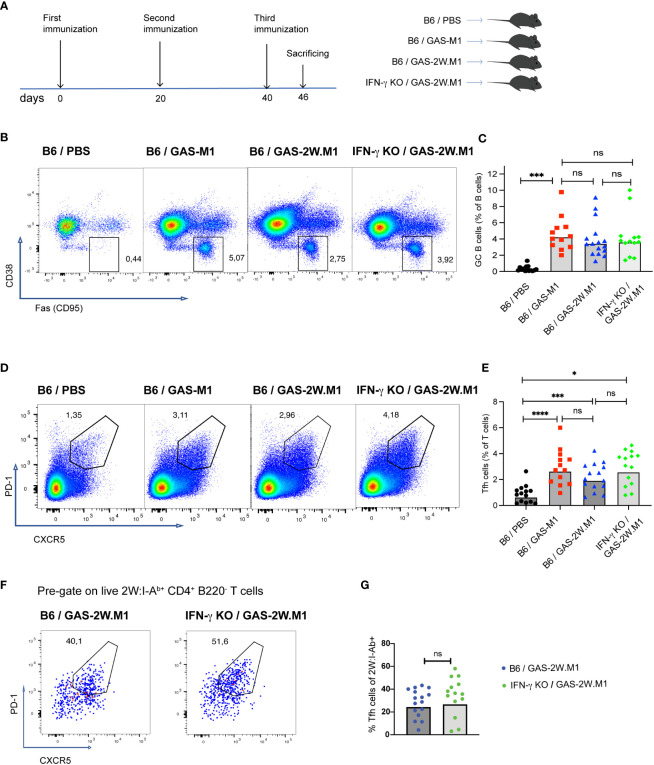
IFN-γ deficiency does not reduce GC B or Tfh cell expansion following GAS-2W.M1 immunization. B6 and IFN-γ KO mice were immunized three times with HK GAS-M1 (B6, n=12) or HK GAS-2W.M1 (B6, n=16; IFN-γ KO mice, n=13) as described in **Figure 1**. Control B6 mice received 3 x PBS only (n=14). Animals were sacrificed seven days after last immunization and the percentages of GC B and Tfh cells in the draining inguinal lymph node were determined by flow cytometry. **(A)** Experimental study design. **(B, C)** Percentage of CD38^-^ CD95^+^ GC B cells after gating on live B220^+^ B cells. Representative results **(B)** and pooled data **(C)**. **(D, E)** Percentage of PD-1^+^ CXCR5^+^ Tfh cells after gating on live CD4^+^ B220^-^ T cells. Representative results **(D)** and pooled data **(E)**. **(F, G)** Percentages of PD-1^+^ CXCR5^+^ Tfh cells among 2W:I-A^b^ tetramer-binding CD4^+^ T cells in GAS-2W.M1-immunized wt and IFN-γ KO mice. Representative plots after gating on 2W:I-A^b+^ CD4^+^ B220^-^ T cells **(F)** and statistical analysis of pooled data **(G)**. Pooled results **(C, E, G)** show individual mice and geometric mean percentages. Statistical significance was analyzed by one-way ANOVA with Tukey’s multiple comparisons test **(C, E)** or by unpaired t-test **(G)**. *p<0.05, ***p<0.001 and ****p<0.0001. ns, not significant.

As GC B cells and Tfh cells appear to be important for the protection conferred by GAS-2W.M1 immunization (see **Figure 2B**), we next determined how these cellular subsets develop in GAS-2W.M1 immunized IFN-γ deficient and sufficient animals, respectively (**Figure 5A**). In agreement with the requirement to immunize multiple times to induce antibody responses and protective immunity against GAS-M1, there was no expansion of CD95^+^ CD38^-^ B220^+^ GC B cells in any of the mouse strains after primary immunization with either GAS-M1 or GAS-2W.M1 (**Figure S2**). For all three immunized groups, GC B cells became detectable after secondary immunization and increased further after the third dose (**Figure S2**). After three doses of HK GAS-2W.M1, GC B cell expansion occurred to the same extent in IFN-γ sufficient and deficient mice, also comparable to the percentage of GC B cells observed in B6 mice immunized with HK GAS-M1 (**Figures 5B, C**). Likewise, Tfh cells, identified as CXCR5^+^PD-1^+^ Th cells, had developed to the same extent in mice immunized with GAS-M1 and GAS-2W.M1 and were not affected by the absence of IFN-γ following GAS-2W.M1 immunization (**Figures 5D, E**). The percentage of CXCR5^+^PD-1^+^ Tfh cells among 2W:I-A^b^-binding CD4 T cells did also not differ between IFN-γ deficient and sufficient mice (**Figures 5F, G**). As expected, CD4 T cells binding to the 2W:I-A^b^ tetramer were not detected in unimmunized or GAS-M1-immunized wt mice (data not shown). Therefore, and in line with our results on the total GAS-specific serum IgG responses (see **Figure 1C**), the insertion of the immunodominant CD4 Th cell epitope 2W in the N-terminal part of the M1 protein does not lead to expanded GCs, and neither is the reduced protection observed in IFN-γ deficient animals after GAS-2W.M1 immunization associated with a reduced expansion of GC B cells.

## 4 Discussion

In this study, we demonstrate that the inability of a wt M1 expressing GAS strain to induce protective immunity in a sc immunization model, can be overcome by the insertion of the immunodominant Th cell peptide 2W into the N-terminal part of the M1 protein. While this modification did not lead to a measurable alteration in the magnitude of the IgG response against the whole GAS-M1 bacterium, GAS-2W.M1 immunization induced a moderately elevated (1 log) total IgG response against the M1 protein, including the HVR domain. Moreover, GAS-2W.M1 immunization generated a specific increase in GAS.M1-specific antibodies of the IgG2c subclass. Protective immunity conferred by GAS-2W.M1 relied on a T cell-dependent antibody response and the cytokine IFN-γ. Consistent with IFN-γ representing a main switch factor for IgG2c in the mouse, the failure to establish protective immunity in IFN-γ deficient mice was associated with reduced specific IgG2c responses. Our results thus imply that modifying the Th cell response against the M protein of GAS is sufficient to convert a non-protective to a protective response against subsequent infections, in turn indicating that Th cell responses against the native M protein are poorly protective.

Important roles for IFN-γ in protective immunity against GAS have been demonstrated before. For example, in a murine model of systemic infection, innate myeloid cells produce high levels of IFN-γ that is necessary for protection (23), and in humans increased levels of serum IFN-γ correlate with IgG1 and IgG3 dominated antibody responses and protection from GAS infection (20). Moreover, and in consistence with our observations, it has previously been shown in a murine skin infection model that wt GAS induced low levels of IFN-γ, but that pretreatment with IL-12 increased IFN-γ and enhanced protection (44). We observe an IFN-γ-dependent increase of specific IgG2c antibodies that correlate with protection. In mice, IgG2c represents the IgG subclass that most efficiently fixes complement (IgG1 and IgG3 in humans) and display higher bactericidal and opsonophagocytic activity than IgG1 (24). Given the important role of IFN-γ in promoting IgG2c isotype switching, we propose that the heightened IgG2c levels contributes to the IFN-γ-dependent protection observed after immunization with GAS-2W.M1. Interestingly, a recent study suggests that Th cell-derived IFN-γ contributes to protection in a murine model of malaria, not by affecting the quantity but the quality of the adaptive immune response, as ablation of IFN-γ in CD4 T cells specifically resulted in deficient IgG2c isotype switch and diminished control of the parasite during the chronic stage of infection (45).

Immunization of B6 mice with the recombinant GAS-2W.M1 strain also resulted in increased levels of IgG against the native M1 protein and its HVR. As no changes in total IgG levels against M1 or the HVR were observed in the IFN-γ-deficient animals, the GAS-2W.M1-driven increase in M1 and HVR IgG levels observed in the B6 mice (see **Figure 1D, E**) is likely disconnected from the IFN-γ-dependent protection conferred by the recombinant strain. It should however not be excluded that increased IgG levels specific for particular epitopes within the M1 protein may be involved also in this context.

The requirement for insertion of immunodominant Th cell epitopes into microbial proteins for generation of protective immunity upon infection or vaccination has been observed previously. The vaccine candidate J8, containing 12 aa from the conserved region of the M protein, was inefficient at inducing protective immunity in mice unless immunodominant T cell epitopes were provided through conjugation with diphtheria toxoid (46). Moreover, introducing T-cell epitopes into the vaccine vector Modified vaccinia Ankara virus (MVA) using ovalbumin (OVA) mediated increased vaccine-induced IFN-γ^+^ CD4 and CD8 T cell responses, and enhanced its protective capacity towards heterologous (non-OVA) antigens (47). Along similar lines, the impact of antigenic protein alterations on adaptive immune function is commonly exploited both in the infection and cancer vaccination fields, where modification of the MHC anchor residues of presented peptides (heteroclitic peptides) can be used to alter the interaction with the cognate TCR, leading to *e.g.* increased IFN-γ responses in specific CD8^+^ T cells (48, 49). Thus, multiple studies indicate that protein antigens can be modified to confer altered interactions with the TCR, leading to the transformation of qualitatively non-protective into protective T cell-dependent responses. If this model of protection (quality rather than quantity) does indeed rely on the intrinsic functions of a particular antibody subclass, it constitutes a principally different protection mechanism than that posed for antibodies directed against the M protein HVR, which are expected to disrupt an essential virulence function of this particular protein domain (50, 51). Of note, the quantitative difference in antibodies directed towards distinct domains of the M protein that comprises the mechanism of antibody immuno-subdominance (14) underlines the importance for the pathogen to protect such a domain and its function. It is tempting to speculate that the absence of IFN-γ-promoting Th cell epitopes in the GAS M protein also has evolved as an immune evasion mechanism, allowing the bacterium to direct the host immune system to generate non-bactericidal (*i.e.* not complement-fixing) antibody responses. This would comprise yet another M protein-mediated escape mechanism evolved by GAS, in addition to the already well-known mechanisms of antigenic variation and subdominance described specifically for the HVR domain (14, 15).

Our experimental system does not permit substantiated conclusions regarding whether IFN-γ is important during the immunization or active infection phase. It should be noted however, that the GAS-M1.2W strain used in this study, generated a TGF-β- and IL-6-dependent Th17 response required to limit the bacterial load in an intranasal infection model (29, 52). Similarly, a strain of *Listeria monocytogenes* designed to secrete a 2W-containing protein predominantly induces IFN-γ^+^ Th1 cells upon intravenous infection, but Th17 dominated responses after infection via the nasal mucosa (53). These observations suggest that promotion of IFN-γ production is not a *de facto* intrinsic characteristic of the 2W peptide, but rather indicates that the addition of a strong T cell epitope allows an enhanced engagement of naïve T cells, whose differentiation is then instructed by the local immune environment. This scenario would also indicate that IFN-γ production during the immunization phase in our experimental system, rather than during the active infection, may be critical for the differentiation of protective T cell responses. Interestingly, this has similarly been proposed in a system where IFN-γ-dependent antibody-mediated protection against pulmonary *Francisella novicida* infection requires the presence of CD4^+^ T cells during immune priming by vaccination, but not during the effector phase after infection (54). Moreover, it has been suggested that the recall memory B cell response that confer antibody-mediated protection against GAS infection after J8 vaccination is underpinned by *de novo* recruited naïve T cells, rather than by vaccine-specific memory T cells (46).

In conclusion, we show that insertion of the immunodominant CD4 T cell epitope 2W into the N-terminal of the M1 protein results in a recombinant GAS strain with an enhanced ability to induce protective immunity against the parental GAS-M1 strain, lacking the 2W epitope, and that this protective response is dependent on IFN-γ and associated with increased and IFN-γ-dependent IgG2c responses against the parental GAS-M1 strain.

## Data availability statement

The raw data supporting the conclusions of this article will be made available by the authors, without undue reservation.

## Ethics statement

The animal study was approved by Lund/Malmö animal ethical committee. The study was conducted in accordance with the local legislation and institutional requirements.

## Author contributions

BJ-L and JP conceived the study. SE performed all experiments with the assistance of TR. SE, BJ-L and JP analyzed data and prepared the manuscript. All authors contributed to the article and approved the submitted version.
