# Supplementary material for: Insertion of an immunodominant T helper cell epitope within the Group A Streptococcus M protein promotes an IFN-γ-dependent shift from a non-protective to a protective immune response
Source: Front Immunol. 2023 Aug 15;14:1241485. doi: 10.3389/fimmu.2023.1241485 (PMC10465795; doi:10.3389/fimmu.2023.1241485)
Supplement: Supplementary file 1 [file DataSheet_1.pdf]

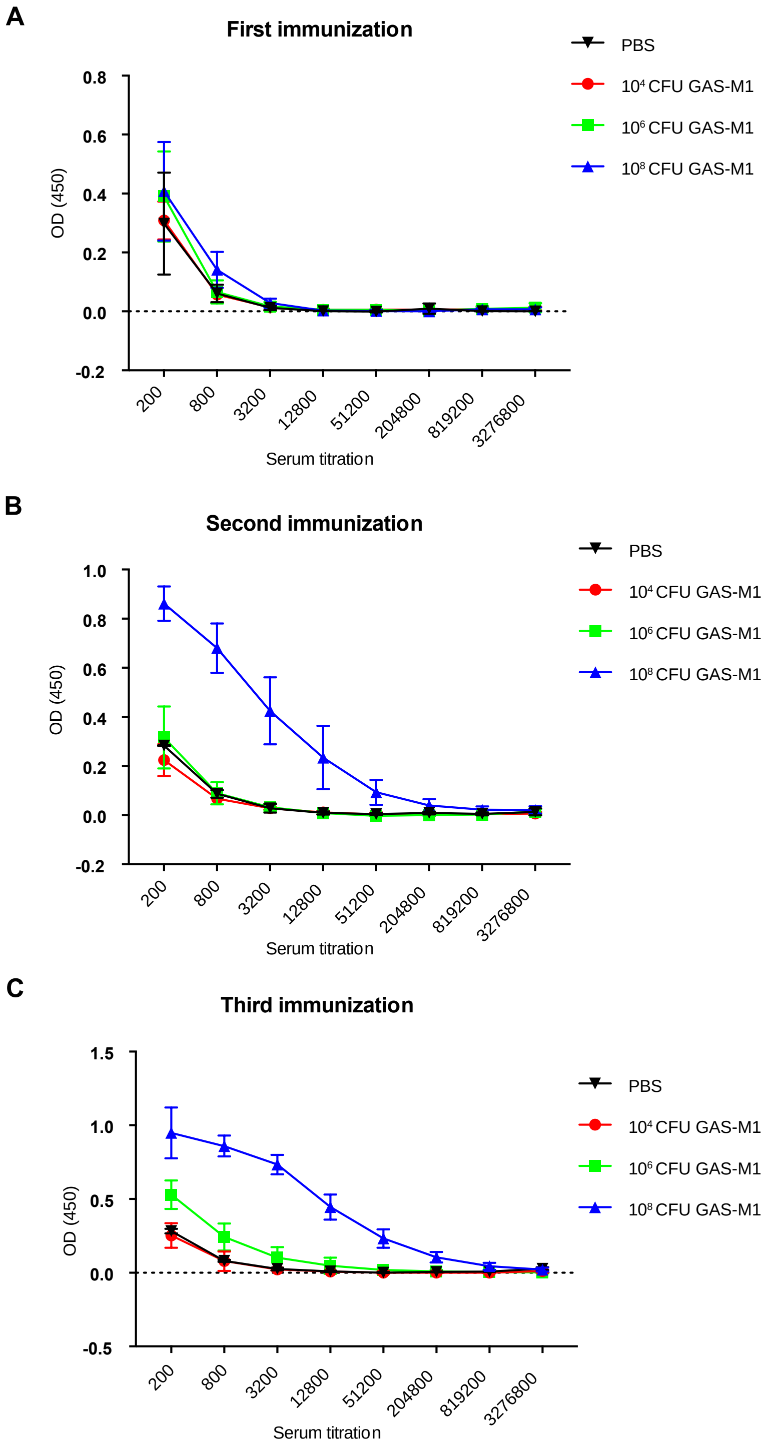

Figure S1. Dose dependent IgG response to HK GAS-M1 immunization. Three groups of B6 mice were injected with increasing doses of HK GAS-M1 according to legend in figure. Control mice received PBS only. Blood was taken from animals 19 days after each immunization and anti-GAS-M1 IgG level was measured by ELISA. ELISA optical densities at 450 nm (OD 450) after the first (A), second (B) and third (C) immunization for serially diluted serum samples are shown (n=4). Results show mean values ± standard deviation.

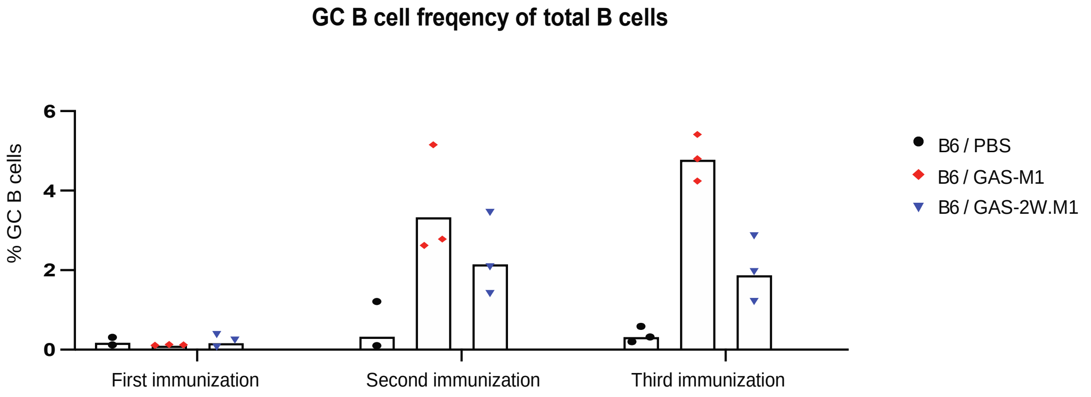

Figure S2. Gradual increase in percentage of GC B cells following repeated immunization with HK GAS-M1 and GAS-2W.M1. Mice were immunized three times sc with HK GAS-M1 (n=9) or GAS-2W.M1 (n=9) as described in figure 1. Control mice received 3 x PBS only (n=7). Percentage of $CD95^{+}$ $CD38^{-}$ GC B cells among total live $B220^{+}$ B cells in draining inguinal lymph nodes was determined by flow cytometry seven days after each immunization.
